# An active ingredient from the combination of Corydalis Rhizoma and Paeoniae Radix Alba relieves chronic compression injury-induced pain in rats by ameliorating AR/Mboat2-mediated ferroptosis in spinal cord neurons

**DOI:** 10.3389/fphar.2025.1558916

**Published:** 2025-03-25

**Authors:** Ze-Ming Wang, Xiao-Hong Wei, Gui-Yang Xia, Lin-Nan Zhou, Jin-Yu Li, Sheng Lin

**Affiliations:** ^1^ School of Chinese Materia Medica, Beijing University of Chinese Medicine, Beijing, China; ^2^ Key Laboratory of Chinese Internal Medicine of Ministry of Education and Beijing, Dongzhimen Hospital, Beijing University of Chinese Medicine, Beijing, China

**Keywords:** chronic pain, chronic compression injury, ferroptosis, AR, Mboat2, extract of Corydalis Rhizoma and Paeoniae Radix Alba, anti‐inflammatory, analgesic effect

## Abstract

**Introduction:**

A combination of Corydalis Rhizoma (the dried tuber of *Corydalis yanhusuo* W.T. Wang) and Paeoniae Radix Alba (the root of *Paeonia lactiflora* Pall.) has been traditionally employed for analgesia. However, the underlying pharmacological mechanisms have not been clarified. The aim of the present study was to investigate the anti-inflammatory and analgesic effects of YB60, the 60% ethanol elution fraction derived from the combination of Corydalis Rhizoma and Paeoniae Radix Alba, and the explore the underlying mechanism.

**Methods:**

Lipopolysaccharide-induced cellular inflammation model and chronic compression injury (CCI) rat model were used to study the anti-inflammatory and analgesic effects of YB60. Proteomics and molecular biology experiments were applied to explore the potential analgesic mechanism of YB60.

**Results:**

The results demonstrated that YB60 significantly decreased inflammatory cytokine levels both in cellular models and rat serum, while concurrently elevating pain thresholds in CCI rats. Proteomic analysis indicated that YB60 could upregulate the expression of Membrane Bound O-Acyltransferase Domain Containing 2 (Mboat2), a newly confirmed marker of ferroptosis. Furthermore, YB60 prevented ferroptosis in the spinal cords of CCI rats. Western blotting and immunofluorescent dual staining further revealed that YB60 increased the expression of Mboat2 and its upstream signaling molecule Androgen receptor (AR). Results in PC12 cells *in vitro* showed that YB60 reversed the downregulation of AR and Mboat2, and ameliorated ferroptosis induced by Erastin, while knockdown of AR eliminated the above effects of YB60.

**Conclusion:**

These findings indicated that YB60 exerted its analgesic effect by inhibiting ferroptosis in spinal cord neurons via modulation of the AR/Mboat2 pathway.

## Introduction

Chronic pain, primarily characterized by persistent pain and unpleasant emotional experiences, encompasses neuropathic pain (NP) and inflammatory pain (IP) (van Hecke et al., 2013). According to the Centers for Disease Control and Prevention, chronic pain has been estimated to affect approximately 20.4% of American adults (Dahlhamer et al., 2018). With a high global prevalence, it severely diminishes quality of life and imposes substantial economic burdens on individuals and society. While the current treatment landscape relies heavily on non-steroidal anti-inflammatory drugs (NSAIDs) and opioids, these medications are associated with considerable toxic side effects (Machado et al., 2017; Corder et al., 2018). Aspirin, one of the most commonly used NSAIDs, remains a mainstay prescription for chronic pain management. Despite its effectiveness, it carries risks of side effects, including liver injury and gastrointestinal hemorrhage (Biglione et al., 2020; Pardutz and Schoenen, 2010). Morphine, a widely prescribed opioid, demonstrates high efficacy in pain management. Nevertheless, its use is complicated by the risks of addiction and potential for misuse (Breault et al., 2024). Currently, the treatment of chronic pain still faces many challenges, particularly in addressing chronic NP and IP. Thus, exploring the underlying mechanisms of chronic pain and creating innovative, effective pain-relief medications are critically important.

Traditional Chinese medicine (TCM) has proven highly effective in managing chronic pain. Treatments such as herbal remedies, acupuncture, and massage can significantly reduce pain symptoms and enhance the quality of life of patients (Guo et al., 2024; Pan et al., 2022). Both Corydalis Rhizoma, the dried tuber of *Corydalis yanhusuo* W.T. Wang, and Paeoniae Radix Alba, the root of *Paeonia lactiflora* Pall., are commonly used for analgesia (Liu et al., 2021b; Sui et al., 2016). In TCM, a combination of Corydalis Rhizoma and Paeoniae Radix Alba is featured in numerous formulas with analgesic effects, such as *Shu-Gan-Zhi-Tong Pill*, *Man-Gan-Jie-Yu Capsule*, *Qi-Zhi- Wei-Tong Granule*, *Gu-Jin Pill*, *Mo-Luo-Dan*, and *Wu-Jin Tablet* (Chinese Academy of Sciences’ Traditional Chinese Medicine Disease Medication Database, http://www.organchem.csdb.cn). In recent years, a growing body of research has indicated that Corydalis Rhizoma and Paeoniae Radix Alba possess distinct advantages in the management of chronic pain. Both L-tetrahydropalmatine from Corydalis Rhizoma and paeoniflorin from Paeoniae Radix Alba exhibit potent analgesic properties (Liu et al., 2021a; Ma et al., 2023). However, the mechanisms underlying the analgesic effect have not been clarified.

Ferroptosis, a recently identified form of cell death, is closely linked to pathophysiological processes in various illnesses, including tumors, neurological disorders, ischemia‒reperfusion injuries, renal injuries, and hematological disorders. In the context of rats with CCI, the occurrence of ferroptosis within the spinal cord and dorsal root ganglion tissues is observed to be tightly associated with the emergence of pain. This type of cell death in the spinal cord and dorsal root ganglia has been implicated in the heightened pain responsiveness observed in CCI rodents, as reported in reference (Wan et al., 2023). Traditionally, the detection of ferroptosis has relied on pathways that are largely under the control of Glutathione peroxidase 4 (GPX4) or Ferroptosis Suppressor Protein 1 (FSP1) (Bersuker et al., 2019). An emerging piece of research has uncovered the AR/Mboat2 pathway as an unprecedented regulator of ferroptosis, demonstrating that Mboat2 has the potential to suppress this form of cell death through the modification of cellular phospholipid architecture (Liang et al., 2023). Despite these findings, the role of the AR/Mboat2 pathway in the progression of ferroptosis within the CCI rat model remains an open question.

The sciatic nerve CCI model serves as a standard approach for investigating NP. Surgically constricting the sciatic nerve in rodents elicits symptoms akin to those observed in human neuropathic pain, such as heightened sensitivity to touch and heat. Since its introduction in 1988, this model has garnered attention for its ability to mimic the symptoms of neuropathic pain and inflammation, providing a dual-purpose platform for assessing both NP and IP (Bennett and Xie, 1988). By developing a rat model with CCI, this study aimed to investigate the anti-inflammatory and analgesic effects of YB60, the 60% ethanol elution fraction derived from the combination of Corydalis Rhizoma and Paeoniae Radix Alba, as well as its underlying mechanisms, with a focus on AR/Mboat2-mediated ferroptosis.

## Materials and methods

### Experimental animals

Healthy adult male Sprague–Dawley rats weighing between 180 and 200 g were used in this study; all the rats aged 6–8 weeks were sourced from Beijing Vital River Laboratory Animal Technology Co., Ltd. All the rats were maintained in an SPF-grade facility with unrestricted access to food and water under a 12 h light/dark cycle. The experimental procedures were approved by the Animal Ethics Committee of Beijing University of Chinese Medicine (Ethics No. BUCM-2023041004-2096). All animal procedures followed the guidelines of the National Institutes of Health (United States).

### Drugs and reagents

Corydalis Rhizoma (Batch No. 2012007) and Paeoniae Radix Alba (Batch No. 21090601) were obtained from Dongzhimen Hospital. Their plant names have been checked with “*The Plant List*” (http://www.theplantlist.org) and accessed via the website. Herbarium samples were deposited at the Key Laboratory of Chinese Internal Medicine of the Ministry of Education and Beijing (herbarium no. 20220618). Indomethacin (M026722) was purchased from Sigma. Lipopolysaccharide (LPS) (L8880) and a CCK-8 kit (CA1210) were acquired from Solarbio. The nitric oxide (NO) detection kit (S0021S) was from Beyotime. Kits for tumor necrosis factor-α (TNF-α, INS302058R), interleukin-1β (IL-1β, INS302869R), and interleukin-6 (IL-6, INS302856R) were purchased from Inselisa. Malondialdehyde (MDA) assay kits (E-BC-K028-M, E-BC-F007) and total iron assay kits (E-BC-K772-M) were obtained from Elabscience. Reactive oxygen species (ROS) ELISA kits (RK15283) were purchased from ABclonal.

### Extraction and separation of active components from the combination of corydalis Rhizoma and Paeoniae Radix Alba

A total of 10 kg of dried Corydalis Rhizoma slices and 10 kg of dried Paeoniae Radix Alba slices were evenly divided into 20 portions. Each portion, consisting of 500 g of Corydalis Rhizoma and 500 g of Paeoniae Radix Alba mixed in a 1:1 ratio, which is commonly used in clinical applications, was soaked in 10 times its volume of distilled water for 24 h. Ultrasonic extraction was performed three times for 1 h per session. The filtered liquids were combined and subjected to column chromatography over macroporous adsorption resin D101 (0.3 mm–1.25 mm, A832686, Shanghai Macklin Biochemical Co., Ltd.), loaded into a glass column (12 × 120 cm) with a bed volume of 6 L. The ultrasonic extracts were directly separated via the column and sequentially eluted with water, 20% ethanol, 40% ethanol, 60% ethanol, and 95% ethanol. The eluates from each fraction were collected and evaporated to dryness via a rotary evaporator, and the yield of each component was calculated.

### High Performance Liquid Chromatography

The extracts of Corydalis Rhizoma and Paeoniae Radix Alba were analyzed using High Performance Liquid Chromatography (HPLC). Chromatographic separation was performed on a YMC-Triart C18 ExRS (4.6 mm × 250 mm, 5 μm) column maintained at 30°C. The injection volume was 5 μL, and the mobile phase flow rate was 1.0 mL/min. A gradient elution program was utilized, employing a mixture of 0.1% trifluoroacetic acid (A) and acetonitrile (B) as follows: 0–20.00 min, 5%–40% B; 20.01–30.00 min, 40%–95% B; and 30.01–35.00 min, 95%–100% B.

Fingerprint analysis of YB60 by HPLC was achieved via a CAPCELL-PAK-UG 120 C18 (4.6 mm × 250 mm, 5 μm) column. The injection volume was 5 μL. The gradient elution employed 0.1% trifluoroacetic acid (A) and acetonitrile (B) as the mobile phase at a flow rate of 1.0 mL/min. The gradient program was as follows: 0–5.00 min, 5%–20% B; 5.01–25.00 min, 20%–40% B; 25.01–29.00 min, 40%–95% B; and 29.01–35.00 min, 95%–100% B (Wu et al., 2022).

### Cell viability detection

The extracted components were formulated into drugs at specific concentrations. Log-phase cells were seeded into 96-well plates, with 30,000 RAW 264.7 cells per well and 10,000 BV2 cells per well. Once the cells had adhered, the medium was replaced, and the cells were treated with various drug concentrations for 24 h. The viability of the cells in each group was then assessed via a CCK8 assay (CA1210, Solarbio Life Sciences, Beijing, China). The solution in each well was removed and substituted with 100 µL of a mixture containing CCK8 reagent and complete culture medium, mixed at a ratio of 1:10. The plates were subsequently incubated at 37°C for 1 hour. The absorbance was measured using a microplate reader (PerkinElmer, United States) at 450 nm. Highly toxic components were excluded, and suitable dosages were established (Cui et al., 2021).

### Establishment of the cell inflammation model

The cell seeding method followed the same procedure as previously described. Once the cells adhered, they were divided into different groups and subjected to various interventions. To establish a cell inflammation model, all the groups were treated with 1 μg/mL LPS (L8880, Solarbio Life Sciences, Beijing, China) for 24 h simultaneously. After this period, the cell supernatant was collected to measure the NO content, and the cell lysates were collected to assess the levels of inflammatory factors (Karthikeyan et al., 2021).

### Nitric oxide detection assay

Griess reagent 1 and Griess reagent 2 from the NO detection kit (S0021S, Beyotime, Wuhan, China) were equilibrated to room temperature prior to use. A standard solution series was prepared through serial dilution with complete culture medium to achieve final concentrations of 0, 1, 2, 5, 10, 20, 40, 60, and 100 µM. Aliquots (50 µL/well) of both cell culture supernatant and diluted standard solutions were dispensed into a 96-well plate under light-protected conditions. Following sample loading, 50 µL of Griess reagent 1 was introduced to each well, with 50 µL of Griess reagent 2 being added subsequently. Absorbance measurements were conducted at 540 nm using a microplate reader (Li et al., 2022).

### Mass spectrometry analysis of YB60

YB60 was analyzed using ultra-performance liquid chromatography coupled with UPLC-Q-Exactive Orbitrap-MS in both positive and negative ionization modes. Chromatographic separation was performed on a Waters BEH C18 column (2.1 × 100 mm, 1.7 μm) at 40°C. The mobile phase consisted of 5% aqueous methanol (A) and methanol with 0.1% formic acid (B), delivered at 0.3 mL/min with a multi-step gradient: 15%–27% B (0–12 min), 27%–35% B (12–13 min), 35%–41% B (13–15 min), 41%–60% B (15–16 min), 60%–67% B (16–22 min), 67%–75% B (22–25 min), 75% B (25–26 min), and 75%–100% B (26–27 min). Mass spectrometry parameters included: m/z scan range 150–2000, sheath gas (50 Bar), auxiliary gas (10 Bar), capillary temperature (350°C), and ion source temperature (320°C). Ionization voltages were set to 4 kV (positive) and 3.5 kV (negative). The system operated in full MS/data-dependent MS^2^ mode (Top 5 precursor ion selection). MS1 spectra were acquired at 70,000 resolution, while MS2 fragmentation utilized 17,500 resolution with stepped normalized collision energies (NCE 20, 40, 60) (Wu et al., 2022).

### Detection of inflammatory factors in cell lysates

The levels of IL-1β, IL-6, and TNF-α in cell lysates were measured using ELISA kits. Cells from the culture plates were collected and resuspended in phosphate buffered saline (PBS) (100 μL/well). Cell lysates were performed through repeated freeze-thaw cycles, followed by centrifugation at 1168 *g* for 15 min to collect the supernatant for analysis. Standards and samples (50 μL/well) were added to the plates. Horseradish peroxidase (HRP)-conjugated detection antibodies (100 μL) were added, and the plates were incubated at 37°C for 60 min. The liquid was discarded, and the plates were washed. Subsequently, 50 μL of substrate A and 50 μL of substrate B were added to each well, followed by incubation at 37°C for 15 min. The reaction was terminated by adding stop solution, and Optical density (OD) was measured at 450 nm (Zhang et al., 2023).

### Preparation and grouping of the CCI model

Dose selection for CCI rat interventions was guided by our preliminary experimental results, with YB60 administered at 25 mg/kg, 50 mg/kg, and 100 mg/kg to establish a therapeutic gradient (Supplemetary Figure S1). The rats were randomly assigned to the following groups: the Sham, CCI, low-dose YB60 (25 mg/kg, CCI + YB60-L), medium-dose YB60 (50 mg/kg, CCI + YB60-M), high-dose YB60 (100 mg/kg, CCI + YB60-H), Corydalis Rhizoma and Paeoniae Radix Alba mixed at a 1:1 ratio (2.08 g/kg, CCI + YB), and indomethacin (M026722, Sigma, MO, United States) positive control (5 mg/kg, CCI + Indo) groups. Following the methods of Bennett and Xie (Bennett and Xie, 1988), the rats were anesthetized with 1% pentobarbital sodium. The right thigh muscles were bluntly separated to expose the sciatic nerve, which was then ligated at four points with 4–0 chromic gut sutures spaced 1 mm apart. In the sham group, only the sciatic nerve was exposed without ligation. Starting on the third day post-surgery, drugs were administered daily via gavage for 7 consecutive days, after which samples were collected. All groups followed the principles of randomization and blinding.

### Measurement of mechanical and thermal pain thresholds

Pain thresholds were performed at preoperative day (−1) and postoperative days 1, 3, 5, 7, and 9. The mechanical pain threshold was evaluated via an electronic von Frey pain measurement instrument (Ahlborn, ALMEMO2490-1A). The experiment was performed in a quiet setting. Rats were placed in transparent metal grid cages and given 30 min to acclimate and calm down before testing commenced. The right hind paw of each rat was stimulated using an electronic Vonfrey device. The withdrawal response was recorded immediately upon paw retraction. Each rat underwent three measurements, with a minimum interval of 10 min between tests. The mean value of the three measurements was calculated and used as the daily mechanical pain threshold for that rat. The thermal pain threshold was measured via a hot plate (IITC Life Science). Prior to the test, rats were acclimated to a serene environment, with a 50°C hot plate ready for use. Once the rats were tranquil, they were placed into a transparent cylinder on the hot plate tester, and a timer was initiated. The timer was halted, and the response was documented when the rats showed signs of discomfort, such as withdrawing, lifting, or licking their paws. The rats were promptly removed to avoid any harm. Each rat underwent this process three times, with a minimum of 10 min between each trial. The average of these three measurements was calculated to determine the rat’s thermal pain threshold for that day (Zhang et al., 2019).

### Detection of inflammatory factors in serum

Blood samples were obtained from the rats via abdominal aorta puncture. After collection, the blood was centrifuged at 1609 *g* at 4°C for 15 min. Serum obtained from supernatant extraction was stored at −80°C and thawed to room temperature before detection (Arafat et al., 2023). The contents of TNF-α, IL-1β, and IL-6 in serum of rats were detected by corresponding ELISA kit. The procedure followed the steps were described in the ELISA section.

### Ultrastructure of spinal dorsal horn

Mitochondrial damage within the L4-L6 spinal dorsal horn regions of the rats was revealed using transmission electron microscopy (TEM). Samples were first fixed using glutaraldehyde. After fixation, the tissues underwent dehydration through a series of ethanol solutions and were then cut into ultrathin sections. These sections were stained with uranyl acetate and lead citrate to enhance contrast. The mitochondrial morphology was detected by HT-7500 transmission electron microscope (Hitachi, Co. Japan) by blinded pathologists (Dang et al., 2022).

### Proteomic analysis

Spinal cord samples were obtained from the Sham group, CCI group, and CCI + YB60-H group. After the rats were anesthetized and sacrificed, the spinal cords were rapidly extracted, snap-frozen in liquid nitrogen, and stored at −80°C. TMT-labeled quantitative proteomics and LC‒MS/MS were used to identify proteins and screen for differential proteins. A Kyoto Encyclopedia of Genes and Genomes (KEGG) analysis of these proteins was performed (Wei et al., 2022).

### Detection of ferroptosis-related biochemical markers

Spinal cord samples were homogenized, and the supernatant was collected to measure the levels of Fe^2+^, MDA, and ROS. The concentrations of Fe^2+^, MDA and ROS were determined by the iron assay kit (E-BC-K772-M, Elabscience, Wuhan, China), MDA assay kit (E-BC-K028-M, E-BC-F007, Elabscience, Wuhan, China) and ROS assay kit (RK15283, ABclonal, Wuhan, China), following the protocol described in these references (Yang et al., 2022; Wei et al., 2025).

### Protein expression assay by western blot analysis

Protein samples were obtained from the spinal cords of rats in the Sham, CCI, and CCI + YB60-H groups. Following tissue homogenization, protein extraction was performed to measure the protein levels of AR and Mboat2. Proteins isolated from spinal cords or cellular extracts were solubilized in RIPA buffer supplemented with protease and phosphatase inhibitors. The protein concentration was quantified using the BCA Protein Assay Kit (MA0082, MeilunBio). Protein lysates (50 µg) from each experimental group were separated by 10% SDS-PAGE and subsequently transferred to a PVDF membrane. Following a blocking step with 5% nonfat dry milk, the blots were incubated with the primary antibodies against AR (1: 1000, Ab133273, Abcam), Mboat2 (1: 500, Novus NBP2-83185), and GAPDH (1:20000, A19056, ABclonal) overnight at 4°C. The membrane was then rinsed with TBST and incubated with the corresponding secondary antibody for 2 h at room temperature. The immunoreactive bands were visualized using an ECL detection system with an ECL kit (MA0186, MeilunBio), and the band intensities were analyzed using BandScan software (Dang et al., 2022).

### Establishment of AR knockdown models

The AR gene (NM_012502.2) was sourced from the cDNA library of GeneChem (Shanghai, China). Plasmid vectors for expressing shRNA targeting the AR gene sequence (GCT​GCT​CCG​CAG​ACA​TTA​AAG) and a negative control (TTC​TCC​GAA​CGT​GTC​ACG​T) were synthesized and cloned and inserted into the GV493 vector with a BsmBI site (purchased from GeneChem, Shanghai). After the recombinant plasmid was confirmed by DNA sequencing, it was transfected into *E. coli*, and the ShAR plasmid was extracted via a Qiagen plasmid extraction kit (Hilden, Germany). PC12 cell transfection experiments were subsequently performed via Lipo3000 to establish AR knockdown models (Chen et al., 2021a).

### Double immunofluorescence labeling

AR and Mboat2 were found to be colocalized with c-Fos, with a particular emphasis on the dorsal horn area of the rat spinal cord. The paraffin-embedded tissue sections were first dewaxed. Next, they were immersed in citrate buffer and heated in a water bath at 99°C for antigen retrieval. After cooling to room temperature, the sections were rinsed with PBS. The tissue areas were outlined using a histochemical pen, and blocking solution was applied for 30 min to prevent nonspecific binding. A mixture of two primary antibodies from different sources was prepared and applied to the sections, which were then incubated overnight at 4°C in a humidified chamber. Following PBS washes, the corresponding secondary antibody was added, and the slides were incubated in the dark at room temperature for 50 min. After another PBS wash, DAPI staining solution was applied, and the slides were incubated in the dark for 10 min to label nuclei. The sections were then treated with an autofluorescence quenching agent for 5 min and rinsed under running water for 10 min. Finally, the slides were mounted using an anti-fade mounting medium and examined under a fluorescence microscope (Ding et al., 2022).

### Statistical analysis

Statistical analyses were conducted via *Prism* 9.0.0 software. Pain thresholds were evaluated via *two-way ANOVA*, and comparisons for other measurements between groups were made via *one-way ANOVA*. The results are expressed as the means ± standard deviation (SD), with *P* < 0.05 indicating statistical significance.

## Results

### Among the 5 extracts of YB, YB60 exhibited optimal effects on cell viability and NO inhibition induced by LPS in RAW264.7 and BV2 cells

We separated YB into 5 extracts via macroporous resin with varying concentrations of ethanol (0%, 20%, 40%, 60%, and 95%), named YB0, YB20, YB40, YB60, and YB95. HPLC was performed separately for each extract (Supplemetary Figure S2A). Then, they were used to treat RAW 264.7 cells and BV2 cells to observe their effects on cell viability. YB95 was excluded due to its high toxicity; YB0 and YB20 exhibited no cytotoxicity up to a concentration of 400 μg/mL; YB40 showed no cytotoxicity within 100 μg/mL; and YB60 demonstrated no cytotoxicity at concentrations below 200 μg/mL (Supplemetary Figures S2B, C).

In conjunction with the CCK8 assay results, we assessed the inhibitory influence of low, medium, and high concentrations of YB0, YB20, and YB60 on the NO content induced by LPS at concentrations of 50, 100, and 200 μg/mL, respectively. With the exception of YB0, all the fractions demonstrated varying levels of NO suppression, with a clear dose-dependent trend, with YB60 being the most effective (Supplemetary Figures S2D, E).

### YB60 was primarily composed of alkaloids and monoterpenoids

Fingerprint analysis of YB60 was shown in Supplemetary Figure S3A. To further clarify the main components of YB60, UPLC-Q-Exactive Orbitrap-MS was employed for detailed analysis. A total of 46 components were identified, including 27 from Corydalis Rhizoma, primarily alkaloids, and 19 from Paeoniae Radix Alba, mainly monoterpene glycosides (Supplemetary Figure S3B; Supplemetary Table S1).

### Interaction of YB60 with the inflammation cell model

**FIGURE 1:**
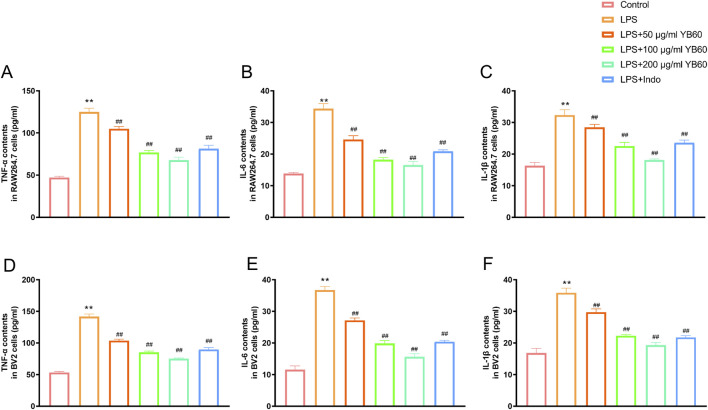
YB60 alleviated inflammation induced by LPS in RAW 264.7 and BV2 cells. A-C. The effects of YB60 on TNF-α **(A)**, IL-6 **(B)**, and IL-1β **(C)** contents in RAW 264.7 cells. D-F. Effects of YB60 on TNF-α **(D)**, IL-6 **(E)**, and IL-1β **(F)** levels in BV2 cells. The error bars represent the SD. ^**^ *P* < 0.01 vs. the control group; ^##^ *P* < 0.01 vs. the LPS group.

To investigate the effect of YB60 on inflammation, we used LPS-induced inflammation cell model, and detected the contents of TNF-α, IL-1β, and IL-6. The results revealed that the levels of TNF-α, IL-1β, and IL-6 induced by LPS increased significantly in both RAW 264.7 and BV2 cell lines. However, pretreatment with different concentrations of YB60 significantly inhibited the release of TNF-α, IL-1β, and IL-6 in a dose-dependent manner (Figures 1A–F). These results indicate that YB60 has a good inhibitory effect on inflammation.

### YB60 demonstrated effective anti-inflammatory and analgesic properties in CCI rats

**FIGURE 2:**
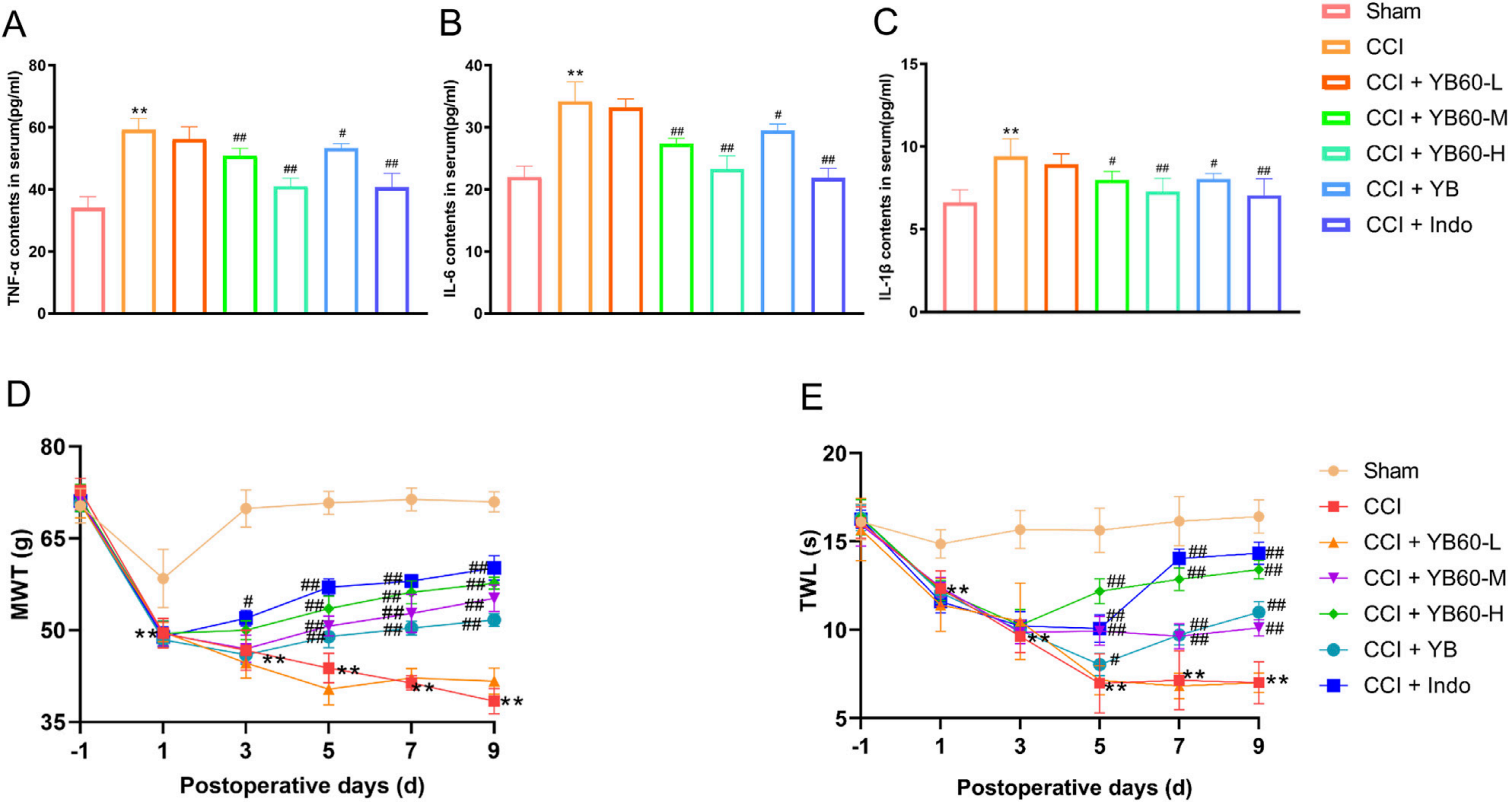
YB60 has strong anti-inflammatory and pain-relieving effects on CCI rats. A-C. Serum levels of TNF-α **(A)**, IL-6 **(B)**, and IL-1β **(C)** in each group of rats. **(D)** MWT in each group of rats. **(E)** TWL in each group of rats. The error bars represent the SD. ^**^ *P* < 0.01 vs. the sham group. ^#^ *P* < 0.05 vs. the CCI group. ^##^ *P* < 0.01 vs. the CCI group.

To further assess the anti-inflammatory properties of YB60, we evaluated the serum inflammation levels in CCI rats. As shown in Figures 2A–C, after the operation, the contents of inflammatory factors were significantly elevated in the CCI group compared with those in the Sham group (*P* < 0.01). Indomethacin (5 mg/kg), which is commonly used as a positive control for anti-inflammatory and analgesic effects, was administered to validate the efficacy of YB60 (Rukshala et al., 2021). As anticipated, indomethacin significantly reduced the serum levels of inflammatory cytokines in CCI rats (*P* < 0.01). When YB60 was administered, there was a dose-dependent reduction in the levels of inflammatory factors. The YB group also presented a decrease in inflammatory factor levels (*P* < 0.05). These findings suggest that YB60 inhibits the release of inflammatory factors triggered by nerve damage, indicating a potent anti-inflammatory effect.

A rat model of chronic NP was established via sciatic nerve ligation, and the effect of YB60 on the MWT was assessed on the basis of the foot withdrawal response on the ligated side. No significant differences were observed in the baseline hind paw Mechanical withdrawal threshold (MWT) among the rat groups (*P* > 0.05). On the first postoperative day, the MWT in the sham group slightly decreased, and it stabilized after the third postoperative day. On the first postoperative day, a significant decrease in the MWT was observed in all groups except the sham group, confirming that the operation was successful. After the operation, the MWT in the CCI group was significantly lower than that in the sham group. From the third postoperative day, the MWT in the indomethacin group was significantly greater than that in the CCI group (*P* < 0.01). The MWT in the CCI + YB60-H group was significantly greater than that in the CCI group, whereas the CCI + YB60-L group showed no significant improvement, demonstrating a dose-dependent effect. Additionally, beginning on the fifth postoperative day, the MWT in the YB group also significantly increased but remained lower than that in the CCI + YB60-M group (Figure 2D). These findings suggest that YB60 effectively suppresses mechanical stimulation pain in CCI rats.

No significant difference was observed in the baseline hind paw Thermal withdrawal latency (TWL) among the different groups (*P* > 0.05). The TWL in the sham group stabilized throughout the observational period. On the first postoperative day, all groups showed a significant decrease in TWL except for the Sham group, confirming that the operation was successful. After the operation, the TWL in the CCI group was significantly lower than that in the sham group. From the fifth postoperative day, the TWL in the indomethacin group was significantly greater than that in the CCI group (*P* < 0.01). YB60 increased the TWL in CCI rats in a dose-dependent manner. Additionally, beginning on the fifth postoperative day, the TWL in the YB group also significantly increased (Figure 2E). These findings suggest that YB60 effectively inhibits pain induced by thermal stimulation in CCI rats.

### YB60 prevented ferroptosis in the spinal cords of CCI rats

**FIGURE 3:**
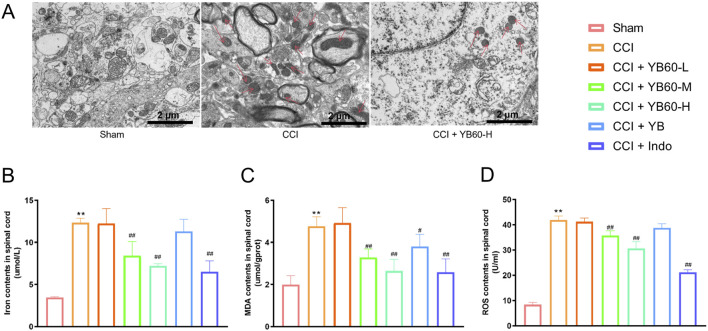
YB60 modulates ferroptosis in spinal cord of CCI rats. **(A)** TEM images of the L4‒L6 spinal cord dorsal horns of Sham, CCI, and CCI + YB60-H rats, with arrows pointing to mitochondria displaying ferroptosis features. B-D. Contents of iron ions **(B)**, MDA **(C)** and ROS **(D)** in various groups, n = 6. The error bars represent the SD. ^**^ *P* < 0.01 compared with the Sham group. ^#^ *P* < 0.05 compared with the CCI group. ^##^ *P* < 0.01 compared with the CCI group.

To determine whether ferroptosis contributed to pain in CCI rats, we assessed related markers. TEM revealed no significant mitochondrial abnormalities in the sham group, whereas the CCI group presented notable mitochondrial crista shrinkage, increased membrane density and reduced mitochondrial volume. Compared with CCI rats, CCI rats treated with 200 mg/kg YB60 presented less mitochondrial damage, with fewer damaged mitochondria and more intact structures (Figure 3A). These ultrastructural observations confirm spinal ferroptosis activation in CCI pathology and its pharmacological suppression by YB60. Moreover, the concentrations of Fe^2+^, MDA, and ROS were significantly greater in the spinal cords of CCI rats than in those of sham rats. YB60 significantly decreased these markers in a dose-dependent manner, whereas indomethacin had no significant effect on these markers (Figures 3B–D). These findings suggest that YB60 prevents ferroptosis in the spinal cords of CCI rats, which may be closely related to the analgesic effect.

### Proteomic analysis also indicated that YB60 exerted analgesic effects on CCI rats through the ferroptosis pathway

**FIGURE 4:**
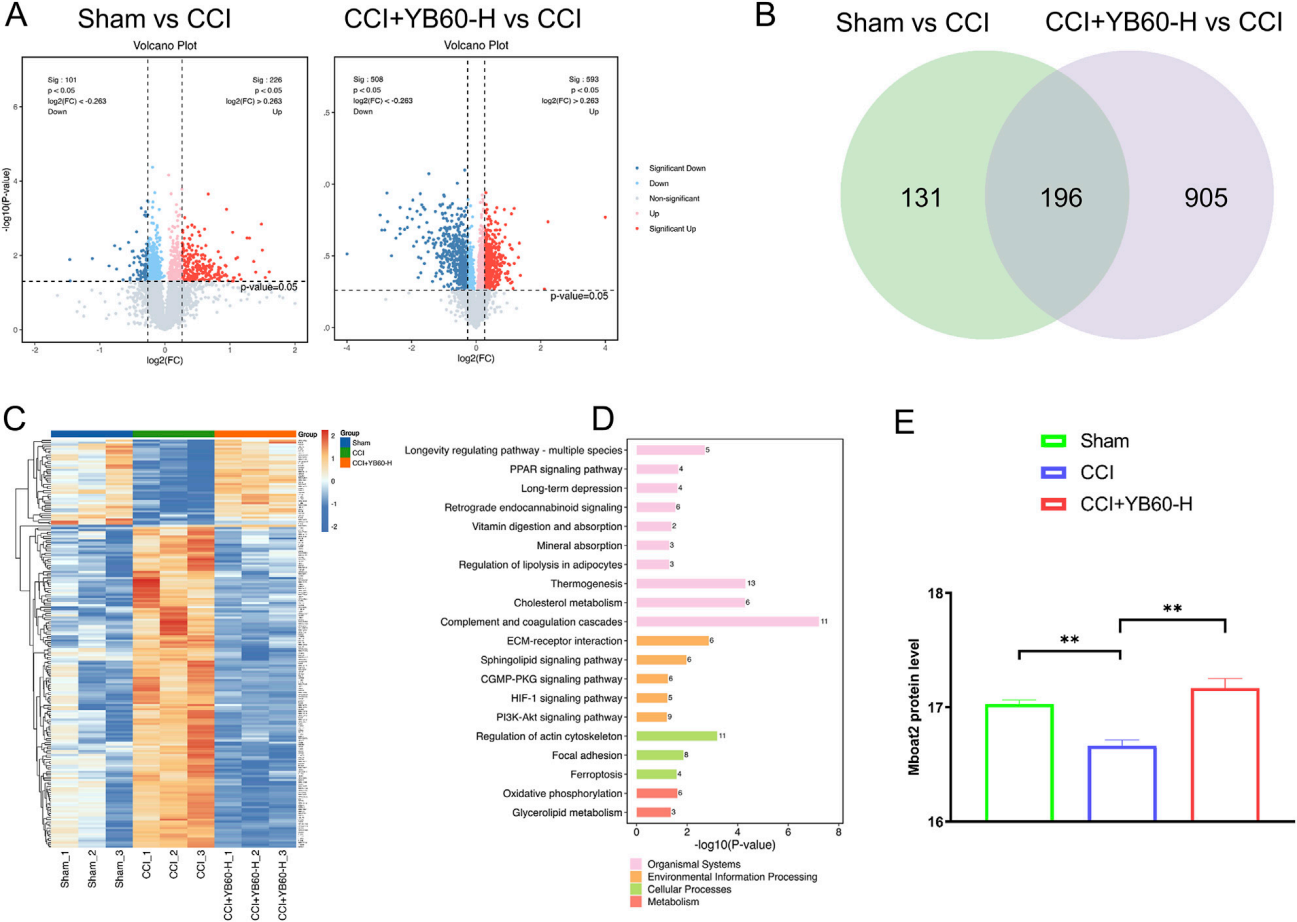
TMT proteomics analysis of the L4‒L6 spinal cord segments in CCI rats. **(A)** Differential protein expression. The volcano plot displays log2 (FC) on the horizontal axis, with values further from zero indicating greater differential expression, right indicating upregulation, and left indicating downregulation. The vertical axis shows -log10(P value), with higher values indicating greater significance. Red and blue dots indicate upregulated and downregulated proteins, respectively, with deeper colors indicating more significant changes, whereas gray dots indicate proteins with p values ≥0.05. **(B)** Venn diagram of the differentially expressed proteins. Different colors denote different groups, with the numbers indicating the count of intersecting and unique proteins for each group. **(C)** Heatmap of differential protein clustering. Proteins are clustered by expression level, with red indicating higher expression and blue indicating lower expression. Each row represents the expression level of each protein across different groups. **(D)** KEGG pathway analysis of intersecting proteins. **(E)** The protein level of Mboat2 in the Sham, CCI and CCI + YB60-H groups. ^**^ *P* < 0.01.

Nine samples from a single TMT batch were analyzed, and 6,147 proteins were identified via LC‒MS/MS. Proteins were filtered on the basis of a score sequence HT > 0, at least one unique peptide, and complete expression values, resulting in 5,520 reliable proteins after median normalization and log2 transformation. We further identified differentially expressed proteins on the basis of a fold change ≥1.2 or a fold change ≤1/1.2 with a p value <0.05. Compared with the Sham group, the CCI group presented 226 upregulated and 101 downregulated proteins, whereas the CCI + YB60-H group presented 593 upregulated and 508 downregulated proteins (Figure 4A). A Venn diagram analysis revealed 196 overlapping differentially expressed proteins between the two groups (Figure 4B). Clustering analysis of these 196 proteins revealed expression patterns across the groups, including signal recognition particle receptor subunit beta (SRP-β), acyl-CoA synthetase long-chain family member 6 (Acsl6), Inhibitor of carbonic anhydrase (Inhca), and Mboat2, which were closely related to the ferroptosis pathway (Figure 4C). To explore the biological pathways involved, a KEGG analysis of these 196 proteins was performed. Among these, long-term depression, amyotrophic lateral sclerosis, ferroptosis, and retrograde endocannabinoid signaling were closely associated with pain (Figure 4D). Consistent with the above-mentioned results, proteomic analysis further confirmed that YB60 effectively prevented ferroptosis from occurring in the spinal cords of rats.

Emerging evidence has established ferroptosis as a critical pathophysiological contributor to chronic pain pathogenesis. Ferroptosis has been observed in the spinal cord and dorsal root ganglion tissues of both CCI rats and rats with complete Freund’s adjuvant-induced inflammatory pain (Wan et al., 2023; Deng et al., 2023b). However, the mechanistic contribution of ferroptosis to CCI-induced hyperalgesia has yet to be fully delineated, representing a critical gap in understanding pain pathophysiology. Recent research identified the AR/Mboat2 pathway as a novel ferroptosis mechanism, with Mboat2 capable of inhibiting ferroptosis by remodeling cell phospholipid structures. This research was published in *Cell* (Liang et al., 2023). Interestingly, our study revealed that Mboat2 protein levels were markedly decreased in CCI rats. However, intervention with YB60 significantly elevated the expression of the Mboat2 protein (Figure 4E). This indicates that YB60 may exert its analgesic effect by modulating ferroptosis through the AR/Mboat2 pathway. Building on insights into this regulatory axis, we focused on AR/Mboat2-regulated ferroptosis, which might be a potential pathophysiological mechanism underlying chronic neuropathic pain induced by CCI.

### YB60 inhibited ferroptosis in the spinal cord neurons of CCI rats by reversing the downregulation of AR and Mboat2

**FIGURE 5:**
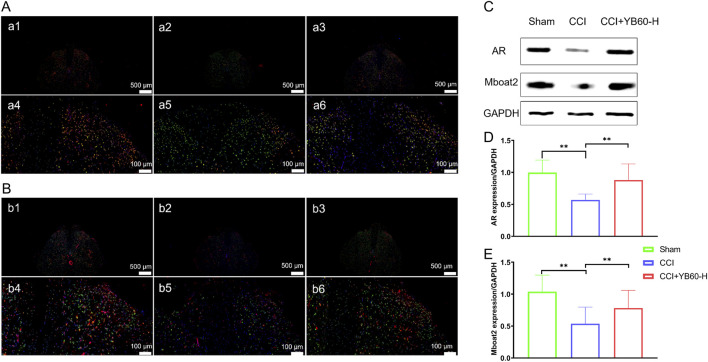
YB60 modulates ferroptosis and the AR/Mboat2 axis in Spinal Cord Neurons. **(A)** Fluorescence double-label staining of AR (green) and c-Fos (red) in the Sham (a1, a4), CCI (a2, a5) and CCI + YB60-H (a3, a6) groups. DAPI (blue) staining highlights nuclei. Scale bars: 500 μm (a1- a3); 100 μm (a4- a6). **(B)** Fluorescence double-label staining of Mboat2 (green) and c-Fos (red) in the sham (b1, b4), CCI (b2, b5) and CCI + YB60-H (b3, b6) groups. DAPI (blue) staining highlights nuclei. Scale bars: 500 μm (b1- b3); 100 μm (b4- b6). **(C)** Expression levels of AR and Mboat2. **(D-E)** Semiquantitative analysis of AR **(D)** and Mboat2 **(E)**, n = 3. The error bars represent the SD. ^**^ *P* < 0.01.

We used immunofluorescence double staining to examine AR and Mboat2, along with the neuronal activation marker c-Fos, with a focus on the dorsal horn of the spinal cord. As shown in Figure 5A, AR was indicated by green fluorescence, c-Fos by red, and their colocalization appears in yellow, with nuclei in blue. Colocalization was observed in the dorsal horns of all the groups. Compared with that in the sham group (a1, a4), colocalization in the CCI group (a2, a5) was significantly lower, whereas it was significantly greater in the CCI + YB60-H group (a3, a6) than in the CCI group. Similar results were observed for the colocalization of Mboat2 and c-Fos (Figure 5B). Compared with that in the sham group (b1, b4), colocalization in the CCI group (b2, b5) was significantly lower, whereas it was significantly greater in the CCI + YB60-H group (b3, b6) than in the CCI group. These findings indicate that AR and Mboat2 are functionally co-expressed in nociceptive-activated dorsal horn neurons, implicating this axis in ferroptosis regulatory mechanisms within spinal nociceptive circuits.

Western blot analysis of L4‒L6 spinal cord segments revealed that Mboat2 expression was significantly lower in CCI rats than in sham rats (Figures 5C, E). In contrast, Mboat2 expression was significantly upregulated in the CCI + YB60-H group compared with the CCI group (*P* < 0.01, Figure 5E), which was consistent with the proteomics findings. AR expression followed a similar pattern (Figures 5C, D), with a significant decrease in the CCI group compared with the sham group (*P* < 0.01) and a significant increase in the CCI + YB60-H group compared with the CCI group (*P* < 0.01). These findings suggest that YB60 inhibits ferroptosis in the spinal cord neurons of CCI rats by reversing the downregulation of AR and Mboat2.

### YB60 inhibited erastin-induced ferroptosis by regulating the AR/Mboat2 pathway in PC12 cells

**FIGURE 6:**
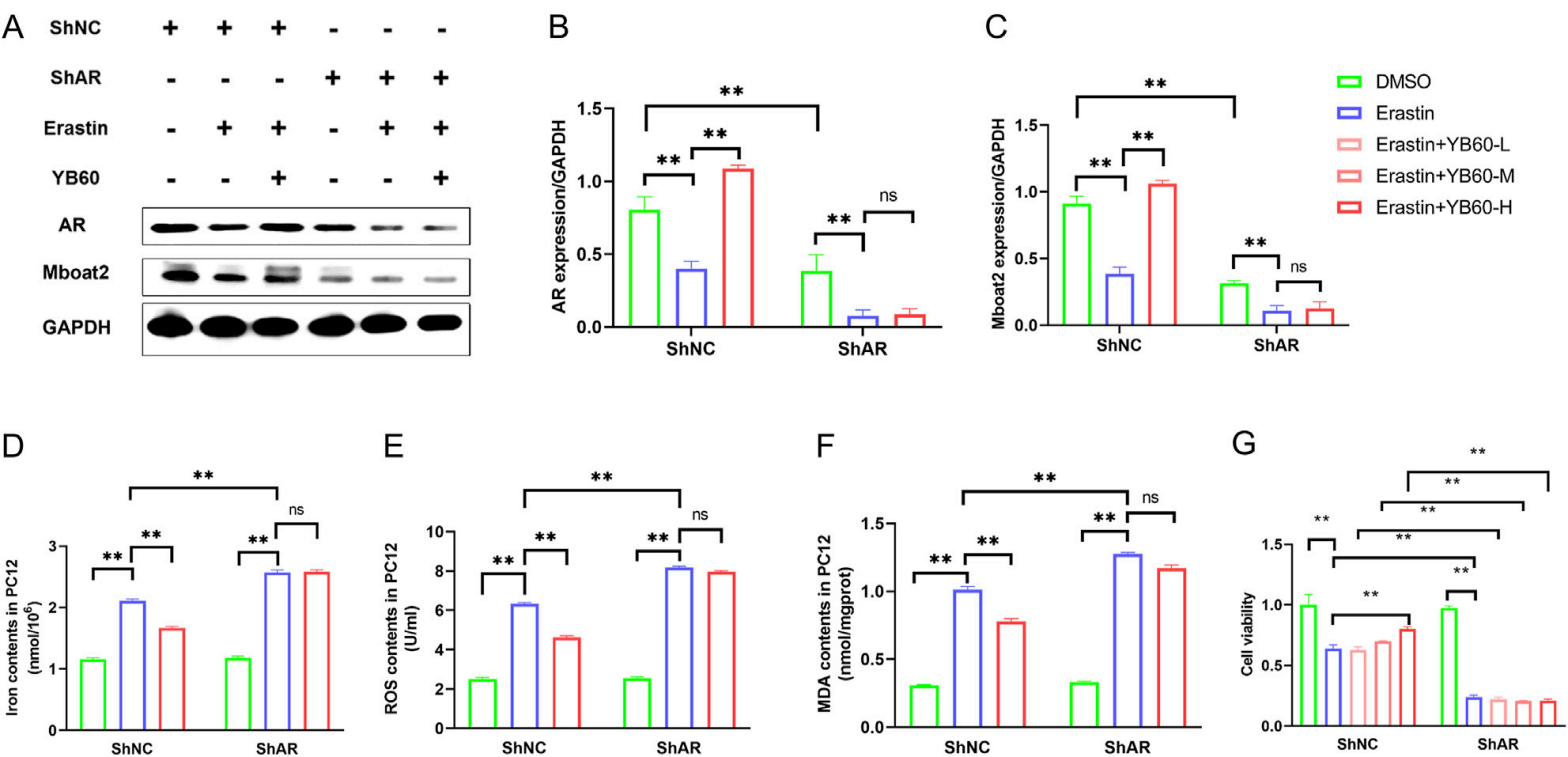
YB60 inhibited Erastin-induced ferroptosis by regulating AR/Mboat2 in PC12 cells. **(A)** Expression levels of AR and Mboat2 in PC12 cells. **(B-C)** Semiquantitative analysis of AR **(B)** and Mboat2 **(C)**, n = 3. D-F. Contents of iron ions **(D)**, ROS **(E)** and MDA **(F)** in various groups, n = 6. **(G)** Viability of PC12 cells. The error bars represent the SD. ^**^ *P* < 0.01.

To explore the regulatory role of YB60 in ferroptosis mediated by AR/Mboat2, we treated AR-knockdown cells (ShAR) and their negative control cells (ShNC) with Erastin and YB60, respectively. As shown in Figures 6A–C, Erastin notably reduced AR and Mboat2 expression in both cell types. YB60 significantly reversed the Erastin-induced downregulation of AR and Mboat2 in ShNC cells, but it had no significant effect on the downregulation in ShAR cells. Similarly, Erastin led to significant increases in iron ion, ROS, and MDA levels in both cell types, which were markedly suppressed by YB60 in ShNC cells. However, after AR knockdown, YB60 had no significant effect on the expression of ferroptosis markers induced by Erastin (Figures 6D–F). Additionally, AR knockdown markedly reduced Mboat2 expression (Figure 6C), further indicating that Mboat2 is a key downstream gene of AR. Compared with ShNC, Erastin caused greater increases in iron ion, ROS, and MDA levels in PC12 cells with ShAR, suggesting that AR-knockdown PC12 cells are more sensitive to Erastin-induced ferroptosis. This finding was also verified, as shown in Figure 6G. These findings reveal that YB60 exerts an inhibitory effect on Erastin-induced ferroptosis through modulation of the AR/Mboat2 pathway.

## Discussion

The present study investigated the anti-inflammatory and analgesic effects of YB60, an active component derived from the combination of Corydalis Rhizoma and Paeoniae Radix Alba, and explored its underlying mechanism, with a particular focus on AR/Mboat2-mediated ferroptosis. Initially, we fractionated the mixture of Corydalis Rhizoma and Paeoniae Radix Alba into distinct extracts. In our earlier screening efforts, the 60% ethanol elution fraction derived from the combination of Corydalis Rhizoma and Paeoniae Radix Alba, which was named YB60, showed promising anti-inflammatory properties, which laid the groundwork for this study. YB60 effectively suppressed the production of NO and the release of TNF-α, IL-1β, and IL-6 induced by LPS in RAW 264.7 and BV2 cells. These key inflammatory mediators are known to exacerbate pain by promoting inflammatory responses and neural sensitivity (Farghaly et al., 2016; Liu et al., 2023). As a result, we selected YB60 for an in-depth study of its anti-inflammatory and pain-relieving properties in CCI rats, uncovering the underlying mechanisms behind its analgesic effects. As shown in the results, YB60 was revealed to exert anti-inflammatory and analgesic effects in CCI rats, effectively reducing inflammatory cytokine levels and significantly enhancing the MWT and TWL of CCI rats. Molecular biology validation, including proteomics and gene interference experiments, confirmed that the analgesic effect of YB60 were closely associated with its regulation of ferroptosis in spinal cord neurons via the AR/Mboat2 pathway.

Numerous studies have shown that inflammation and pain are closely linked both physiologically and pathologically (Ding et al., 2020; Sun et al., 2022). Inflammation, the body’s response to injury or infection, manifests as redness, swelling, heat, pain, and impaired function. Pain, a key symptom of inflammation, arises from the release of mediators like prostaglandins, bradykinin, histamine, and cytokines. These substances sensitize pain-sensing nerves, causing heightened pain responses and pain from normally non-painful stimuli (Sommer et al., 2018). Moreover, inflammation can alter the nervous system, leading to central sensitization, which intensifies pain perception (Ji et al., 2014). Thus, inflammation not only initiates pain but also perpetuates and exacerbates it through intricate molecular and cellular processes, creating a self-sustaining cycle. Conversely, many analgesic drugs achieve their pain-relieving effects by blocking the release of these inflammatory mediators (Haddadi and Rashtiani, 2020; Sultana and Rasool, 2015; Wang et al., 2023). In line with previous findings, our study observed a marked rise in inflammatory cytokines and the development of hyperalgesia in CCI rats. The inflammation likely stemmed from the damaged sciatic nerve, and the subsequent release of inflammatory cytokines contributed to the heightened pain sensitivity in these rats. YB60 reduced the levels of inflammatory cytokines in the serum of CCI rats and increased their pain threshold, which suggested that YB60 possesses anti-inflammatory and pain-relieving properties, similar to those of indomethacin. Mass spectrometry data of YB60 implied that its anti-inflammatory activity was likely mediated by alkaloid and monoterpene constituents. In fact, alkaloids from Corydalis Rhizoma and monoterpenes in Paeoniae Radix Alba have been demonstrated to have remarkable anti-inflammatory effects. For instance, tetrahydropalmatine from Corydalis Rhizoma could reduce the inflammatory response in skin flap necrosis (Yang et al., 2024), while Paeoniflorin in Paeoniae Radix Alba could exert significant anti-arthritis effects (Fei et al., 2019).

Traditionally, the mechanisms underlying chronic pain are mainly attributed to central sensitization and peripheral sensitization. However, cutting-edge research has unveiled a strong link between ferroptosis in spinal neurons and chronic pain, offering a novel insight into the pathological mechanisms of this condition (Wang et al., 2021; Xue et al., 2024; Yang et al., 2023). Research indicates that in chronic pain models, increased iron levels and lipid peroxidation in spinal cord and dorsal root ganglion neurons contribute to abnormal neuronal activity and persistent pain signaling (Chen et al., 2021b). Moreover, imbalances in ferroptosis-related molecules like GPX4 worsen oxidative damage and inflammation in neurons, driving chronic pain progression (Yao et al., 2021). Targeting ferroptosis to reduce lipid peroxidation and neuroinflammation has shown promise in alleviating chronic pain (Zhang et al., 2020). Thus, ferroptosis not only underpins chronic pain mechanisms but also emerges as a promising therapeutic avenue. Through the detection of a range of ferroptosis-related biomarkers and proteomic analysis, we have collectively substantiated the occurrence of ferroptosis in the spinal neurons of CCI rats. YB60 significantly mitigated mitochondrial ultrastructural alterations in CCI rats and reduced iron ion and peroxidation levels, indicating that its analgesic properties may be associated with the inhibition of ferroptosis. Additionally, proteomic findings further indicated that YB60 can modulate proteins related to ferroptosis. Our study has preliminarily elucidated the strong link between chronic pain and ferroptosis. This is consistent with the findings of recent studies. Building on this finding, we have identified a promising new avenue for drug development aimed at treating chronic pain.

As a glutathione-independent ferroptosis suppressor, FSP1 utilizes its reductive capacity to scavenge lipid radicals, thereby mitigating lipid peroxidation and ferroptosis (Doll et al., 2019). GPX4, the canonical glutathione peroxidase family member, prevents ferroptosis through reduction of lipid hydroperoxides (Ursini and Maiorino, 2020). Emerging research highlights that GPX4’s palmitoylation is essential for its ability to suppress ferroptosis (Huang et al., 2025). FSP1 and GPX4 function as separate yet complementary mechanisms, working together to prevent ferroptosis in cells. Recent research has identified the AR/Mboat2 pathway-regulated ferroptosis surveillance mechanism as a novel regulatory pathway for ferroptosis, distinct from the conventional FSP1 and GPX4 pathways (Liang et al., 2023). This mechanism has only been demonstrated in tumor cells, but our study reveals its significant association with the ferroptosis mechanism in chronic pain. YB60 demonstrated AR/Mboat2 pathway modulation sufficient to inhibit neuronal ferroptosis, suggesting therapeutic potential for attenuating CCI-induced hyperalgesia via ferroptosis blockade. Interestingly, our study also reveals that Erastin, a classic ferroptosis inducer (Yang et al., 2020), can induce ferroptosis not only via the GPX4 (Song et al., 2024) and VDACs pathways (Zhang et al., 2015) but also by modulating the AR/Mboat2 pathway in PC12 cells, highlighting Erastin’s versatility in ferroptosis induction.

**FIGURE 7:**
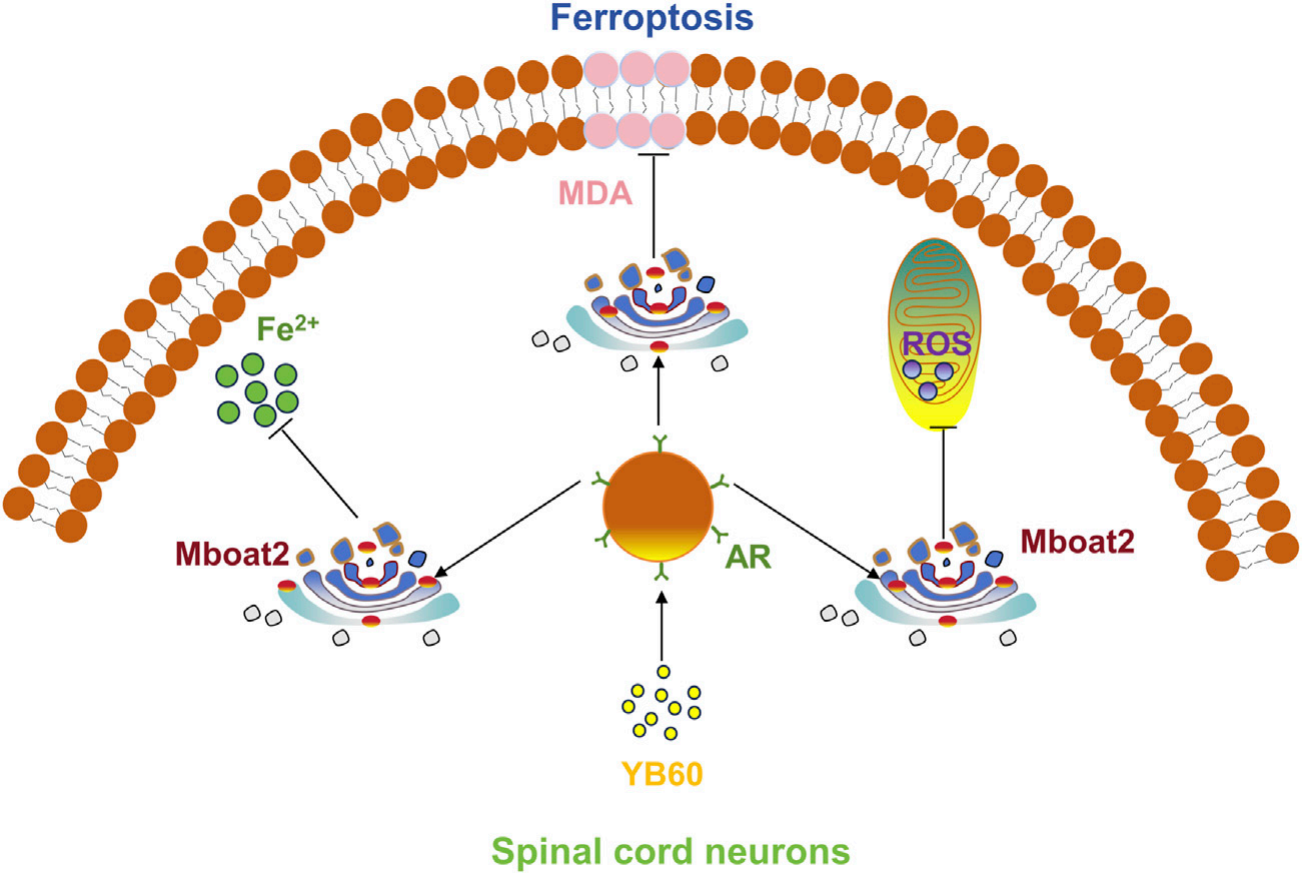
YB60 inhibited ferroptosis in spinal cord neurons by increasing the expression of AR and Mboat2.

YB60 reduced the levels of inflammatory cytokines in the serum of CCI rats and increased their pain threshold. This study suggests that YB60 inhibits ferroptosis in spinal cord neurons by increasing the expression of AR and Mboat2, which contributes to analgesia (Figure 7). Furthermore, our findings indicates that Erastin can trigger ferroptosis in PC12 cells by downregulating the expression of AR and Mboat2, which is independent of the GPX4 and VDACs pathways. Our findings offer a novel perspective on the mechanisms underlying chronic pain and highlight the therapeutic potential of Chinese herbal drugs for managing this condition.

Despite these findings, the study has some limitations. For instance, our study exclusively utilized male rats. Whether ferroptosis contributes to pain in female CCI rats remains unclear. Therefore, further research will be conducted to elucidate this mechanism. Moreover, our research focused on the link between ferroptosis and heightened pain sensitivity but did not explore the potential connection between ferroptosis and inflammation. Notably, growing evidence suggests a close bidirectional regulatory relationship between ferroptosis and inflammatory responses, highlighting the significant potential of targeting ferroptosis in the prevention and treatment of inflammatory diseases (Chen et al., 2023; Deng et al., 2023a). Therefore, further research efforts should be strengthened to advance knowledge in this area.

## Conclusion

The present study elucidated the mechanism underlying the anti-inflammatory and analgesic effects of YB60, the 60% ethanol elution fraction derived from the combination of Corydalis Rhizoma and Paeoniae Radix Alba. Using LPS-induced macrophage cell lines (RAW 264.7 and BV2) inflammatory models alongside a CCI rat model, YB60 was shown to significantly reduce inflammatory cytokine levels and elevate pain thresholds. Proteomic analysis revealed that YB60 upregulated the expression of Mboat2, a ferroptosis marker in spinal cord tissues, and activated AR/Mboat2 signaling pathway, as validated by Western blotting and immunofluorescence. *In vitro* experiments demonstrated that YB60 reversed Erastin-induced suppression of AR/Mboat2 expression and ferroptosis-related damage in PC12 cells, while knockdown of AR abolished these protective effects. These findings indicated that YB60 exerted its analgesic effect by inhibiting ferroptosis in spinal cord neurons via modulation of the AR/Mboat2 pathway. This discovery not only provides novel insights into the modern pharmacological mechanisms of traditional herbal analgesia, but also establishes a theoretical foundation for developing neuropathic pain treatment strategies targeting ferroptosis regulation. Furthermore, the AR/Mboat2 pathway emerges as a potential therapeutic target for novel analgesic drug development. However, the active pharmaceutical ingredient underlying YB60’s therapeutic effects remains uncharacterized. Consequently, we will conduct more in-depth studies in subsequent work. Moreover, our study exclusively utilized male rats. Whether ferroptosis contributes to pain in female CCI rats remains unclear. Therefore, further research will be conducted to elucidate this mechanism.

## Data Availability

The data sources used in this study are available from the corresponding authors upon reasonable request.

## Glossary

Acsl6: Acyl-CoA synthetase long-chain family member 6
AR: Androgen receptor
CCI: Chronic compression injury
FSP1: Ferroptosis Suppressor Protein 1
GPX4: Glutathione peroxidase 4
GSH: Glutathione
HPLC: High Performance Liquid Chromatography
HRP: Horseradish peroxidase
KEGG: Kyoto Encyclopedia of Genes and Genomes
IL-1β: Interleukin-1β
IL-6: Interleukin-6
Inhca: Inhibitor of carbonic anhydrase
IP: Inflammatory pain
LPS: Lipopolysaccharide
Mboat2: Membrane-bound O-acyltransferase domain containing 2
MDA: Malonaldehyde
MWT: Mechanical withdrawal threshold
NO: Nitric oxide
NP: Neuropathic pain
NSAIDs: Non-steroidal anti-inflammatory drugs
OD: Optical density
PBS: Phosphate buffered saline
ROS: Reactive oxygen species
SD: Standard deviation
SRP-β: Signal recognition particle receptor subunit beta
TCM: Traditional Chinese medicine
TEM: Transmission electron microscopy
TNF-α: Tumor necrosis factor-α
TWL: Thermal withdrawal latency
VDACs: Voltage-dependent anion channels
