# Supplementary material for: An active ingredient from the combination of Corydalis Rhizoma and Paeoniae Radix Alba relieves chronic compression injury-induced pain in rats by ameliorating AR/Mboat2-mediated ferroptosis in spinal cord neurons
Source: Front Pharmacol. 2025 Mar 25;16:1558916. doi: 10.3389/fphar.2025.1558916 (PMC11975664; doi:10.3389/fphar.2025.1558916)
Supplement: Supplementary file 1 [file DataSheet1.pdf]

Supplementary materials

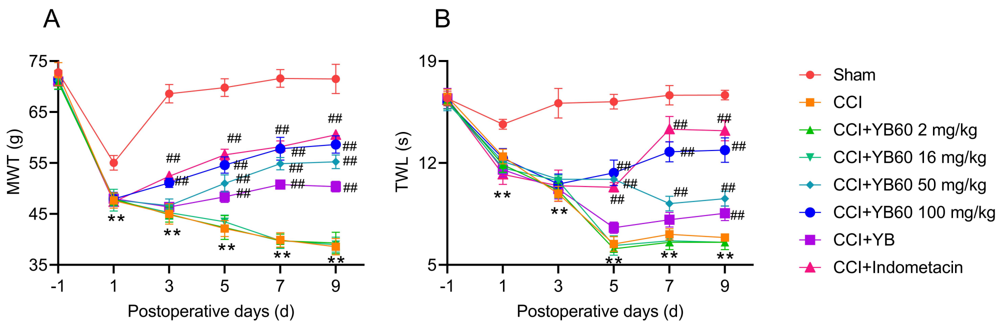


**Figure S1.** Pre-experimental results of Mechanical withdrawal threshold (MWT) and Thermal withdrawal latency (TWL) in rats. A. MWT in each group of rats. n=3. B. TWL in each group of rats. n=3. $^{**}P < 0.01$ vs. Sham group. $^{\#\#}P < 0.01$ vs. CCI group.

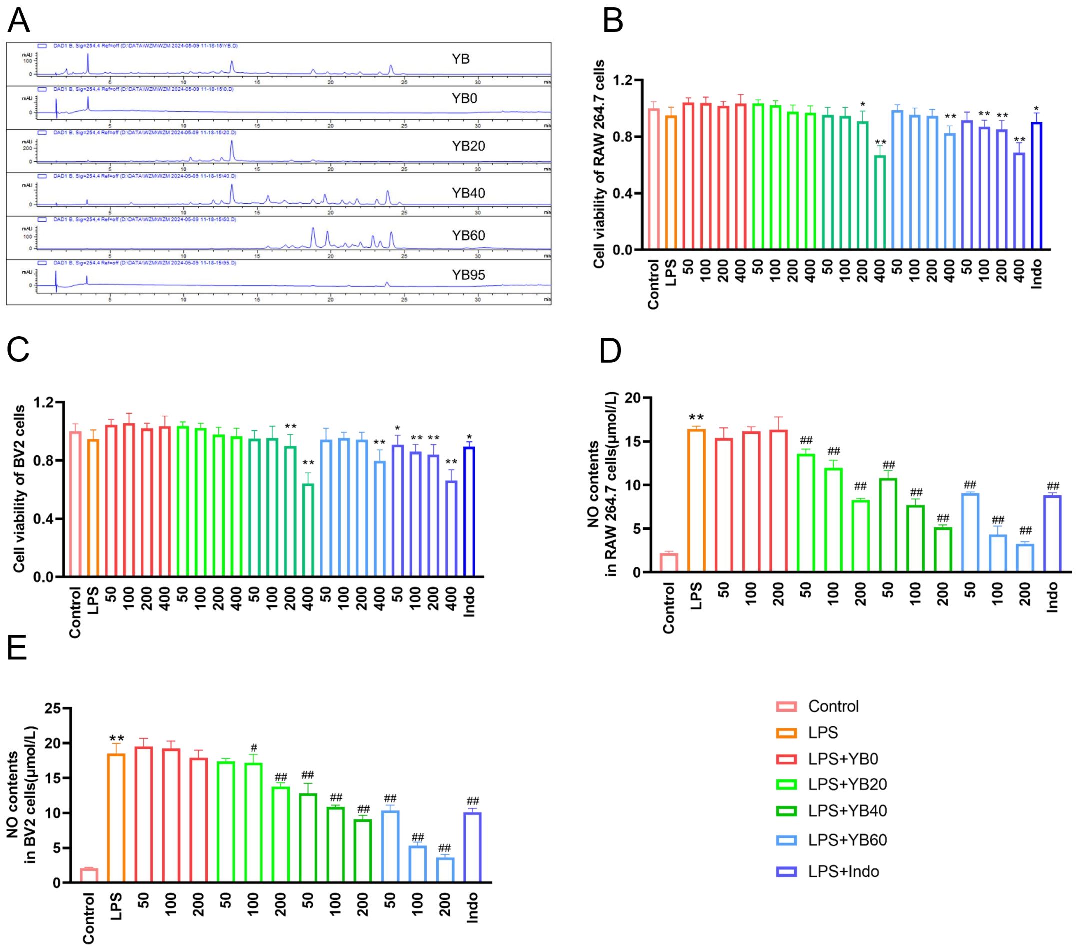


**Figure S2.** A. The HPLC of each subfraction derived from the compatibility of YB. The fractions obtained with different concentrations of ethanol were correspondingly named YB0, YB20, YB40, YB60 and YB95, respectively. B-C. Cell viability of Raw 264.7 cells (B) and BV2 cells (C) treated with different YB fractions under various concentrations. D-E. NO contents in Raw 264.7 cells (D) and BV2 cells (E) treated with different YB fractions under various concentrations.

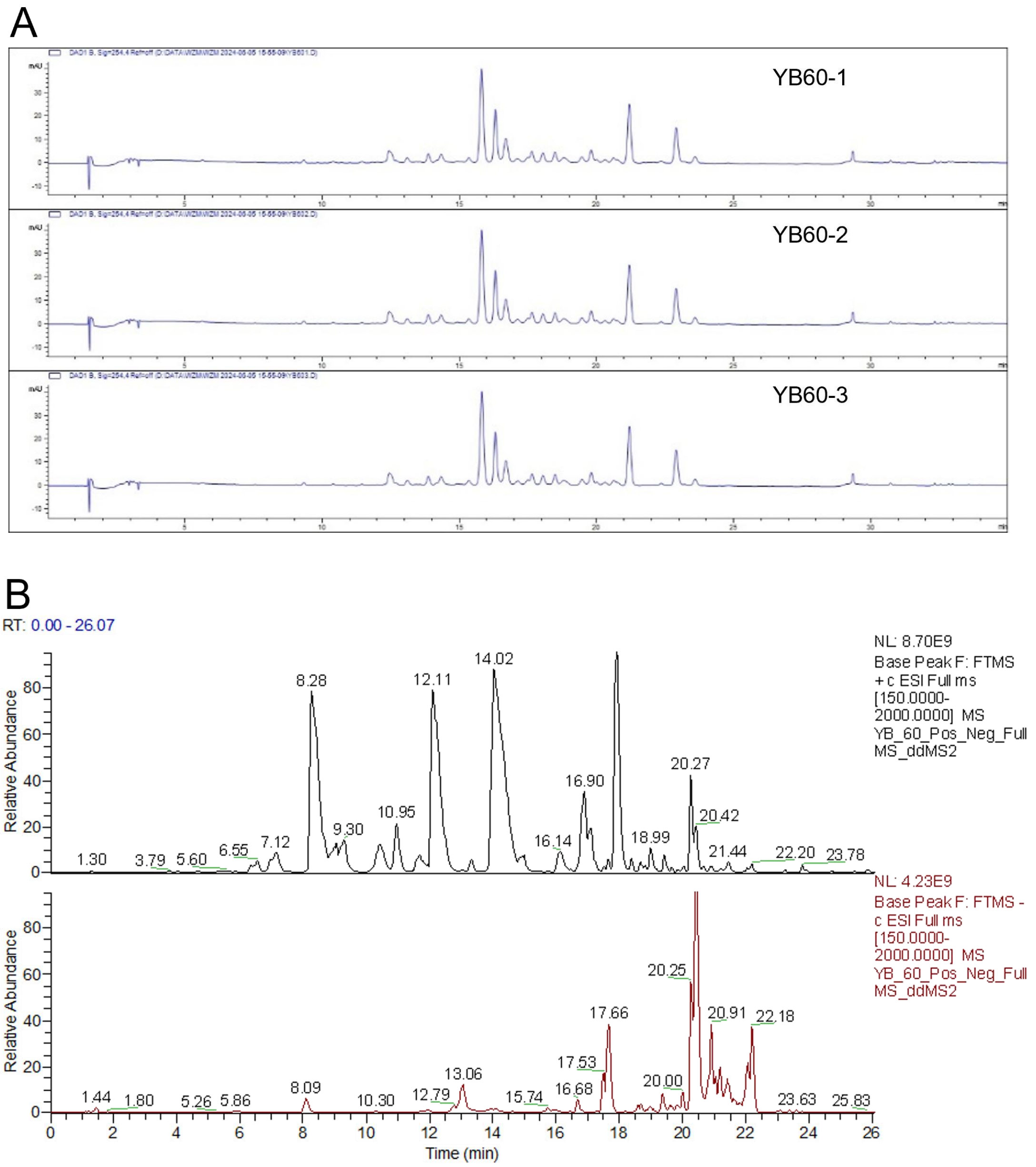


**Figure S3.** A. The fingerprint spectra of YB60. B. The comprehensive ion flow profiles of YB60 captured in both positive and negative ion modes.

**Table S1** The measured values of retention time (RT) and peak mass to charge ratio of each chemical component in the quasi molecular ion peak

| No | Molecular formula | *m/z* | Mass Error | RT (min) | Compound | Fragment ions |
|---|---|---|---|---|---|---|
| 1 | $C_{16}H_{24}O_9$ | $[M+HCOO]^-$/ 405.1411 | 3.46 | 1.63 | Peony ketone-1-O-β -D-glucoside | 359.1352,197.0822, 179.0713 |

| | | | | | | |
|---|---|---|---|---|---|---|
| 2 | $C_7H_6O_5$ | $[M-H]^-$/169.0144 | 1.18 | 1.70 | Gallic acid | 169.0142, 125.0244 |
| 3 | $C_{15}H_{14}O_6$ | $[M-H]^-$/289.0721 | 1.04 | 3.09 | Catechin | 245.0819,203.0708, 151.0400,123.0451, 109.0294 |
| 4 | $C_8H_8O_5$ | $[M-H]^-$/183.0300 | 0.00 | 3.13 | Methyl gallate | 183.0298,168.0065, 124.0166, |
| 5 | $C_{23}H_{28}O_{13}S$ | $[M-H]^-$/543.1183 | 1.11 | 3.74 | Paeoniflorin sulfite | 421.0808,375.0784, 259.0284,213.0227, 121.0294, |
| 6 | $C_{19}H_{21}NO_4$ | $[M+H]^+$/328.1542 | -0.30 | 4.07 | Isoboldine | 328.1536, 297.1122 |
| 7 | $C_{23}H_{28}O_{11}$ | $[M+HCOO]^-$/525.1613 | 0.95 | 5.87 | Albiflorin | 449.1455, 327.1079, 121.0294 |
| 8 | $C_{19}H_{21}NO_4$ | $[M+H]^+$/328.1540 | -0.91 | 6.52 | Scoulerine | 328.1536,178.0860, 163.0625, 151.0752 |
| 9 | $C_{20}H_{23}NO_4$ | $[M+H]^+$/342.1700 | 0.00 | 7.16 | D-Lirioferine | 192.1015,176.0702, 148.0757 |
| 10 | $C_{23}H_{28}O_{11}$ | $[M+HCOO]^-$/525.1608 | 0.00 | 8.13 | Paeoniflorin | 449.1455,327.1079, 165.0556, 121.0294 |
| 11 | $C_{20}H_{23}NO_4$ | $[M+H]^+$/342.1694 | -1.75 | 8.25 | Tetrahydrojatrorrhizine | 342.1694,326.1383, 312.1216,178.0560, 165.0908, 163.0626 |
| 12 | $C_9H_{10}O_5$ | $[M-H]^-$/197.0454 | -0.51 | 8.27 | Ethyl gallate | 169.0142,125.0244, 124.0165 |
| 13 | $C_{30}H_{32}O_{17}$ S | $[M-H]^-$/695.1294 | 1.01 | 8.65 | Galloylpaeoniflorin sulfonate | 259.0281,169.0142, 125.0244 |
| 14 | $C_{23}H_{28}O_{12}$ | $[M-H]^-$/495.1514 | 1.21 | 8.86 | Oxypaeoniflorin | 165.0555, 137.0243 |
| 15 | $C_{20}H_{23}NO_4$ | $[M+H]^+$/342.1697 | -0.88 | 8.95 | Tetrahydrocolumbaminen | 342.1695,326.1388, 178.0860,165.0911, 163.0626 |
| 16 | $C_{21}H_{25}NO_4$ | $[M+H]^+$/356.1854 | -0.56 | 9.19 | Yuanhunine | 356.1851,341.1621, 326.1381,192.1017, 177.0785,165.0909, 150.0675 |
| 17 | $C_{21}H_{23}NO_5$ | $[M+H]^+$/370.1649 | 0.00 | 9.34 | Allocryptopine | 370.1645,352.1180, 322.0712,206.0818, 188.0705, 165.0908 |
| 18 | $C_{20}H_{19}NO_5$ | $[M+H]^+$/354.1335 | -0.28 | 10.39 | Protopine | 354.1327,336.1225, 206.0809,188.0703, 149.0595 |

| | | | | | | |
|---|---|---|---|---|---|---|
| 19 | $C_{22}H_{27}NO_4$ | $[M+H]^+$ /370.2013 | 0.00 | 10.78 | Corydaline | 370.2005,355.1772, 354.1695,340.1540, 165.0909 |
| 20 | $C_{21}H_{25}NO_4$ | $[M+H]^+$ /356.1851 | -1.40 | 10.99 | Corybulbine or Isocorybulbine | 356.1849,341.1624, 340.1540,179.1064, 178.0860, 163.0627 |
| 21 | $C_{19}H_{14}NO_4^+$ | $[M]^+$/320.0914 | -0.94 | 11.63 | Coptisine | 318.0755,290.0804, 277.0728,262.0858, 249.0777, 234.0907 |
| 22 | $C_{21}H_{23}NO_5$ | $[M+H]^+$ /370.1647 | -0.54 | 11.77 | α-Allocryptopine | 206.0812, 188.0703 |
| 23 | $C_{20}H_{21}NO_4$ | $[M+H]^+$ /340.1542 | -0.29 | 11.83 | Nantenine | 324.1220,192.0915, 148.6805 |
| 24 | $C_{30}H_{32}O_{15}$ | $[M-H]^-$/631.1667 | - | 11.89 | - | 631.1665,313.0562, 169.0141, 121.0294 |
| 25 | $C_{21}H_{25}NO_4$ | $[M+H]^+$ /356.1850 | -1.68 | 12.15 | Tetrahydropalmatine | 356.1848,341.1606, 192.1014,177.0782, 165.0908, 150.0673 |
| 26 | $C_{19}H_{17}NO_4$ | $[M+H]^+$ /324.1229 | -0.31 | 12.39 | Tetrahydrocoptisine | 356.1848,341.1606, 192.1014,177.0782, 165.0908, 150.0673 |
| 27 | $C_{30}H_{32}O_{15}$ | $[M-H]^-$/631.1663 | -0.79 | 13.10 | Galloylpaeoniflorin | 613.1552,491.1186, 399.0931,313.0566, 211.0248, 169.0142 |
| 28 | $C_{21}H_{24}NO_4^+$ | $[M]^+$/354.1700 | 0.00 | 13.35 | N-Methylcanadine | 354.1694,190.0860, 165.0909 |
| 29 | $C_{20}H_{21}NO_4$ | $[M+H]^+$ /340.1545 | 0.59 | 13.72 | Tetrahydroberberine | 340.1537,325.1285, 176.0703,165.0908, 135.0443 |
| 30 | $C_{20}H_{19}NO_6$ | $[M+H]^+$ /370.1281 | -1.08 | 13.96 | Taxilamine | 370.1278, 190.0860 |
| 31 | $C_{21}H_{25}NO_4$ | $[M+H]^+$ /356.1850 | -1.68 | 14.03 | D-Glaucine | 295.0958,279.1109, 267.1009,251.0609, 236.0828, 220.0880 |
| 32 | $C_{20}H_{20}NO_4^+$ | $[M]^+$/338.1385 | -0.30 | 14.3 | Jatrorrhizine | 338.1381,307.0829, 279.0887, 265.0734 |
| 33 | $C_{22}H_{28}NO_4^+$ | $[M]^+$/370.2006 | - | 14.68 | N-Methyltetrahydropalmatine | 370.2004,340.1531, 191.0699,176.0705, 165.0908, 135.0437 |
| 34 | $C_{21}H_{23}NO_5$ | $[M+H]^+$ /370.1648 | -0.27 | 14.93 | Cryptopine | 190.0859 |

| | | | | | | |
|---|---|---|---|---|---|---|
| 35 | $C_{20}H_{18}NO_4^+$ | $[M]^+$/336.1227 | -0.89 | 16.07 | Berberine | 336.1123,321.0989,320.0911,306.0754, 304.0960,292.0961, 278.0801 |
| 36 | $C_{30}H_{32}O_{15}$ | $[M-H]^-$/631.1666 | - | 16.71 | - | 631.1664,169.0141, 121.0294 |
| 37 | $C_{21}H_{22}NO_4^+$ | $[M]^+$/352.1539 | -1.14 | 16.80 | Palmatine | 336, 1223, 320.1279,294.1117, 278.0802, 264.1005 |
| 38 | $C_{21}H_{22}NO_4^+$ | $[M]^+$/352.1540 | -0.85 | 17.14 | Dehydrocorybulbine | 337.1303,321.0988, 293.1045, 265.1020 |
| 39 | $C_{24}H_{30}O_{12}$ | $[M-H]^-$/509.1665 | 0.20 | 17.90 | Mudanpioside D | 509.1661, 121.0294 |
| 40 | $C_{22}H_{24}NO_4^+$ | $[M]^+$/366.1695 | -1.37 | 17.91 | Dehydrocorydaline | 351.1458,350.1379, 336.1223,308.1274, 292.0963 |
| 41 | $C_{21}H_{22}NO_4^+$ | $[M]^+$/352.1542 | -0.28 | 18.09 | 13-Methyldehydrocorydalmine | 337.1301,322.1068, 294.1116 |
| 42 | $C_{30}H_{32}O_{13}$ | $[M-H]^-$/599.1771 | 0.17 | 19.33 | Mudanpioside C | 599.1782,551.1583, 447.1298,431.1346, |
| 43 | $C_{30}H_{32}O_{14}S$ | $[M-H]^-$/647.1441 | 0.16 | 19.34 | Benzoylpaeoniflorin sulfonate | 525.1066,259.0281, 121.0294 |
| 44 | $C_{30}H_{32}O_{13}$ | $[M-H]^-$/599.1776 | 1.00 | 19.65 | Benzoyloxypeoniflorin/oxybenzoyl-paeoniflorin | 599.1771,165.0555, 137.0243, 121.0294 |
| 45 | $C_{30}H_{32}O_{12}$ | $[M+HCOO]^-$/629.1874 | 0.64 | 20.48 | Benzoylpaeoniflorin | 553.1726,535.1679, 431.1360,165.0555, 121.0295 |
| 46 | $C_{30}H_{32}O_{12}$ | $[M+HCOO]^-$/629.1877 | 1.11 | 21.43 | Benzoylalbiflorin | 553.1726,535.1679, 121.0295 |
